# The Impact of Phytochemicals in Obesity-Related Metabolic Diseases: Focus on Ceramide Metabolism

**DOI:** 10.3390/nu15030703

**Published:** 2023-01-30

**Authors:** Eunkyeong Kim, Sookyoung Jeon

**Affiliations:** Department of Food Science and Nutrition and the Korean Institute of Nutrition, Hallym University, Chuncheon 24252, Gangwon-do, Republic of Korea

**Keywords:** ceramide, sphingolipid, obesity, metabolic disease, phytochemical

## Abstract

The prevalence of obesity and related metabolic diseases has increased dramatically worldwide. As obesity progresses, various lipid species accumulate in ectopic tissues. Amongst them, ceramides—a deleterious sphingolipid species—accumulate and cause lipotoxicity and metabolic disturbances. Dysregulated ceramide metabolism appears to be a key feature in the pathogenesis of obesity-related metabolic diseases. Notably, dietary modification might have an impact on modulating ceramide metabolism. Phytochemicals are plant-derived compounds with various physiological properties, which have been shown to protect against obesity-related metabolic diseases. In this review, we aim to examine the impact of a myriad of phytochemicals and their dietary sources in altering ceramide deposition and ceramide-related metabolism from in vitro, in vivo, and human clinical/epidemiological studies. This review discusses how numerous phytochemicals are able to alleviate ceramide-induced metabolic defects and reduce the risk of obesity-related metabolic diseases via diverse mechanisms.

## 1. Introduction

The worldwide prevalence of obesity has been alarmingly increasing despite the efforts to prevent and reverse this disease [1]. Obese individuals are at increased risk of insulin resistance and metabolic diseases including type 2 diabetes mellitus (T2DM), non-alcoholic fatty liver disease (NALFD), and cardiovascular disease (CVD), which imposes a significant public health burden [2]. Obesity is multifactorial, resulting from a combination of genetic, epigenetic, physiological, behavioral, and environmental factors [3]. In particular, diet influences the progression and severity of obesity and related metabolic diseases. The Western diet, with high levels of sugar and fat, is known to accelerate obesity development, while the Mediterranean diet, containing high levels of phytochemicals such as polyphenols, can prevent or ameliorate incidences of obesity [4,5,6].

In obesity, a myriad of lipid species accumulate in ectopic tissues and cause lipotoxicity. Of these lipid species, ceramides, a group of sphingolipids, have gained increasing attention due to their metabolic impact. Ceramides are a bioactive lipid species with cell-signaling properties and can disrupt insulin signaling, lipid metabolism, and mitochondrial function [7]. Emerging clinical and preclinical studies reveal that dysregulated ceramides have been implicated in the pathogenesis of obesity-induced insulin resistance and metabolic diseases [8,9].

Ceramide production is increased in response to various stress stimuli such as nutritional overload, inflammation, oxidative stress, and hypoxia [10,11,12]. For example, the consumption of palmitate supplementation or a high-fat diet (HFD) increased circulating ceramide levels while polyunsaturated fat intake decreased circulating ceramides in human subjects [13,14,15]. In addition to macronutrients, intake of various phytochemicals has been associated with the altered ceramide accumulation in the body, as well as with the reduced incidence of metabolic diseases.

In this review, we summarize the ceramide metabolism and the role of ceramide in the pathogenesis of obesity-related metabolic diseases. We highlight the impact of various phytochemicals or related diets on ceramide profiles in body compartments. In addition, we examine whether these changes of ceramide levels affect ceramide-induced lipotoxicity and subsequently mitigate the progression of obesity and metabolic diseases.

## 2. Ceramide Synthesis and Degradation

**Figure 1:**
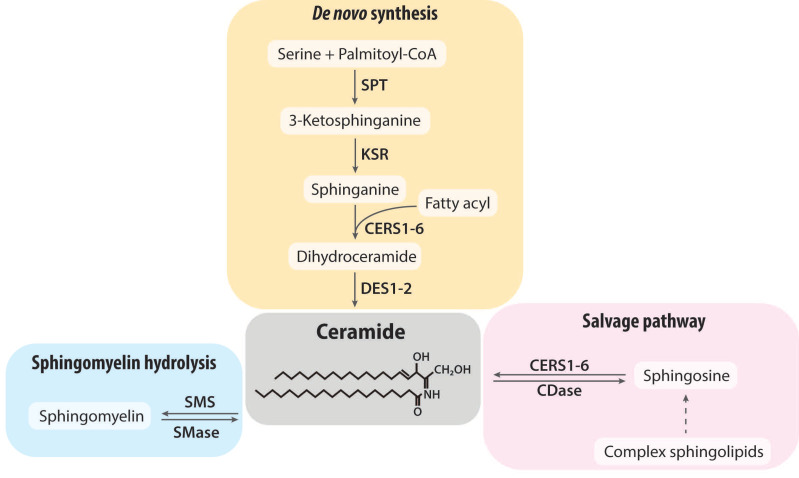
Schematic representation of sphingolipid metabolic pathways that alter ceramide levels. Ceramide can be generated through (1) the de novo synthesis pathway; or (2) sphingomyelin hydrolysis pathway. Complex sphingolipids can also be degraded back to ceramide, which is called (3) the salvage pathway. Abbreviations: SPT, serine palmitoyltransferase; KSR, 3-ketosphinganine reductase; CERS, ceramide synthase; DES, dihydroceramide desaturase; CDase, ceramidase; SMS, sphingomyelin synthase, SMase, sphingomyelinase.

Ceramides contain the basic structure of a sphingoid base linked to a fatty acid of varying chain length. Ceramide resides in the center of sphingolipid metabolism (Figure 1). Sphingolipid metabolism is a complex, compartmentalized, and interconnected intracellular process. There are three major pathways for ceramide formation: (1) the de novo synthesis pathway; (2) the sphingomyelin hydrolysis pathway; and (3) the salvage pathway.

De novo ceramide synthesis is initiated at the endoplasmic reticulum (ER), where serine palmitoyltransferase (SPT) catalyzes the condensation of serine and palmitoyl-CoA to 3-ketosphinganine [16]. This reaction is considered the rate-limiting step of ceramide biosynthesis. Here, SPT can alternatively utilize other fatty acids (e.g., myristoyl-CoA, stearoyl-CoA) or other amino acids (e.g., alanine, glycine) [17,18]. 3-ketosphinganine is reduced to sphinganine by 3-ketoshinganine reductase (KSR). Subsequently, (dihydro)ceramide synthase (CERS) adds fatty acyl chains to generate dihydroceramide, which is a saturated precursor of ceramide. Six CERS isoforms are identified in mammals (CERS1-6) and each isoform utilizes a restricted subset of fatty acyl-CoAs to generate ceramides with defined acyl chains [19]. CERS1 uses mainly C18:0; CERS2 and CERS4 use C22:0 and C24:0; CERS5 and CERS6 have a preference for C16:0. In contrast, CERS3 exhibits a broad substrate specificity. In addition, each CERS isoform has a differential tissue expression pattern. In the last step of de novo synthesis, dihydroceramide is converted to ceramide by dihydroceramide desaturase (DES1-2). Once formed in the ER, ceramides, as well as dihydroceramides, are transported to the Golgi apparatus where they are further converted to complex sphingolipids.

Besides the de novo synthesis, ceramide can be generated via the breakdown of sphingomyelin by the action of sphingomyelinase (SMase). This pathway can rapidly generate ceramides since sphingomyelin is the most abundant sphingolipid in mammals and it needs the activation of a single enzyme, SMase. SMases are distinguished by their pH optima (acid SMase, neutral SMase, alkaline SMase) and subcellular localization [20].

Ceramide can also be generated by catabolizing complex sphingolipids, which is called the salvage pathway or the recycling pathway [21]. Complex sphingolipids such as sphingomyelin and glycosphingolipids are degraded to form ceramide within acidic lysosomes. Then, ceramides are broken down into sphingosine and free fatty acids, which can enter the cytosol. In the cytosol, sphingosine is recycled back to ceramide by the action of ceramidase (CDase).

## 3. Ceramide Metabolism in Obesity-Related Metabolic Diseases

### 3.1. Associations between Ceramide Levels and Obesity-Related Metabolic Diseases

Accumulating evidence demonstrated the deregulation of ceramide metabolism in obese individuals or obese animal models. Several studies have reported that ceramides accumulate in serum or metabolically active tissues (such as liver, adipose tissue, muscle) of insulin-resistant obese patients and non-human primates [22,23,24]. Similarly, increased serum and tissue ceramide levels have been observed in both genetic and diet-induced obese mice [25,26]. In parallel, weight loss by exercise or gastric bypass surgery significantly reduced circulating ceramide species in obese patients [27,28]. In addition to total ceramides, specific acyl-chain ceramide levels (e.g., C16:0, C18:0, C18:1) are shown to be elevated in obese human and animal models. In particular, an increased C16:0-ceramide has been suggested to contribute to obesity development by impairing insulin signaling, fatty acid oxidation, and mitochondrial function [23,29]. Hence, C16:0-ceramide synthesizing enzymes, CerS5 and CerS6, have been implicated in the development of obesity [23,30].

Given that obesity predisposes to various metabolic diseases, ceramide levels have been explored in relation to obesity-related metabolic diseases. First, strong associations between circulating ceramides and the severity of CVD have been reported in various studies [31,32,33]. In specific, high levels of C16:0-, C18:0-, and C24:1-ceramides and low levels of C24:0-ceramides were associated with adverse cardiovascular outcomes and death in humans. Based on these strong associations, ceramide species have been proposed to predict CVD events. In addition to circulating ceramides, myocardial ceramides were elevated in patients with advanced heart failure [34]. In rodent studies, ceramide-lowering strategies have been shown to alleviate the development of several CVDs, proving the link between ceramides and the pathogenesis of CVDs. For example, myriocin, an inhibitor of SPT, prevented the formation of an atherosclerotic lesion in apolipoprotein-E KO mice, resolved hypertension, and improved vascular function [35,36,37].

Numerous human studies have shown strong associations between ceramides, insulin resistance, and T2DM. For example, circulating ceramides are associated with 2-hr post load glucose levels in Australians [38], and with the homeostatic model of insulin resistance (HOMA-IR) in Native Americans [39]. Specifically, higher circulating levels of C16:0-, C18:0-, C20:0-, C22:0-ceramides were associated with a higher risk of developing T2DM [40]. Furthermore, Wigger et al. showed that circulating dihydroceramide, a precursor of ceramide synthesis, may be a potent and sensitive biomarker for T2DM based on two cohort studies [41]. Ceramide accumulation in the liver, adipose tissue, and muscle from human subjects is associated with insulin resistance [22,42,43]. Similarly, increased circulating ceramides are associated with obesity and insulin resistance in high-fat fed mice, *ob/ob* mice, and lipid-infused rats [44,45]. These strong associations are supported by the role of ceramides in impairing insulin signaling, which will be reviewed in the following (Section 3.3).

NAFLD encompasses a spectrum of liver diseases from simple steatosis, non-alcoholic steatohepatitis, and cirrhosis. NAFLD begins with simple steatosis, which is characterized by increased neutral lipids such as triglycerides; ceramides have also been shown to accumulate in the livers of NAFLD patients [46,47]. In addition, serum and hepatic ceramide levels are elevated in various NAFLD rodent models [48]. In particular, insulin resistance, a key pathology of NAFLD progression, is strongly associated with hepatic ceramide deposition in NAFLD patients [42]. Similar to obesity, metabolic pathways involving CerS6 are dysregulated and CerS6-derived C16:0-ceramides are elevated in NAFLD patients and mice [23,29,42]. Taken together, studies in human subjects and animal models have shown that ceramides in serum and tissues are positively associated with obesity and related diseases including CVD, T2DM, and NAFLD.

### 3.2. Accumulation of Ceramide in Obesity

A flurry of studies has suggested several underlying mechanisms that explain elevated ceramide accumulation in obesity. Here, we briefly review the regulation of dysregulated ceramide metabolism and associated factors in obesity. First, increased levels of either exogenous or endogenous precursor substrates (e.g., palmitate) can accelerate ceramide synthesis. The composition of dietary fatty acids can affect the production of ceramides. It was shown that diets rich in saturated fat increased circulating ceramides compared to diets rich in polyunsaturated fat in human subjects [15,49]. In addition, obesity-induced insulin resistance and inflammation exacerbate adipose tissue lipolysis, which can be the source of fatty acid oversupply [50]. These released fatty acids are also incorporated into ceramide formation.

Obesity-mediated chronic inflammation also leads to increase ceramide accumulation. Toll-like receptor (TLR) 4 signaling is required for palmitate-mediated ceramide synthesis, suggesting that ceramide synthesis is facilitated along with inflammatory environment and fatty acid supply [51,52]. Similarly, tumor necrosis factor (TNF) α, a pro-inflammatory cytokine, was also found to stimulate ceramide formation by modulating ceramide de novo synthesis and sphingomyelin hydrolysis [53]. In support of this, other studies reported that circulating ceramide levels were strongly associated with inflammatory cytokines in humans [54,55].

Adiponectin is an adipokine whose levels are negatively correlated with overall adiposity. Adiponectin exerts various beneficial effects such as improving glucose homeostasis by binding to its receptors (AdipoR) [56]. Importantly, adiponectin can stimulate catabolism of ceramide since AdipoR has intrinsic ceramidase function [57,58]. AdipoR overexpression and a synthetic AdipoR agonist (AdipoRon) have been shown to activate ceramidase activity in mice [59,60]. Moreover, adiponectin levels have been reported to negatively correlate with serum and tissue ceramides in insulin-resistant human subjects [46,61]. Taken together, these studies demonstrate that reduced AdipoR-dependent ceramide degradation is a potential mechanism that contributes to increased ceramide accumulation in obesity.

Gut-derived ceramides are another source of ceramide in obesity. Recently, it has been demonstrated that sphingolipids derived from gut bacteria can be absorbed by the host via the portal vein and change host ceramide levels [62]. Bacteroidetes and Chlorobi phylum are the known bacteria that can produce sphingolipids in the gut [63]. Considering that bacterial diversity and abundance are strongly associated with dietary patterns, it is highly possible that obesity-induced bacterial alterations might lead to increase gut bacteria-derived ceramide levels. Of note, altered gut microbiota can also contribute to modulating the intestinal ceramide biosynthesis via farnesoid X receptor (FXR) signaling. FXR is a bile acid-activated nuclear receptor, mainly expressed in the liver and intestine. Jiang et al. showed that gut microbiota regulates a bile acid-intestinal FXR signaling that controls ceramide production in the host [64,65]. The authors have also found that inhibition of intestinal FXR by antibiotics treatment in HFD-fed mice reduced the expression of genes regarding ceramide biosynthesis enzymes and subsequently circulating ceramide levels. Together, gut bacteria may have either direct or indirect impact on ceramide levels in the host.

### 3.3. Cellular Actions of Ceramide Related to Metabolic Disturbances

Ceramides exhibit numerous cellular actions associated with obesity-related metabolic disturbances. Here, we will highlight the ceramides’ actions in insulin signaling, mitochondrial function, and lipid metabolism. Notably, ceramide is known to impair insulin signaling primarily by inhibiting Akt/protein kinase B, a serine/threonine kinase. Ceramide-induced Akt inhibition can lead to activate anabolic pathways and inhibit catabolic pathways, specifically impairing insulin-stimulated glucose uptake and utilization. Ceramide inhibits the activation of Akt via two mechanisms. First, ceramides inactivate Akt by activating protein phosphatase 2A (PP2A) that dephosphorylates both activating residues, Ser^473^ and Thr^308^ [66,67]. Secondly, ceramides inhibit Akt signaling by promoting protein kinase C (PKC) ζ phosphorylation of Akt at the inhibitory site, Thr^34^ [68]. In parallel, various studies demonstrated that genetic or pharmacological inhibition of ceramide production alleviated insulin resistance in insulin-resistant and diabetic mice models [45,69,70,71].

In addition to glucose metabolism, ceramide also influences lipid metabolism. Ceramide inhibits the action of hormone-sensitive lipase (HSL) and then blocks the release of fatty acids from triglycerides via PP2A activation [71]. In addition, ceramide can increase the expression of the fatty acid translocase CD36, and then promote fatty acid passage through lipid bilayers via PKCζ activation [71,72]. Then, ceramide induces the expression of lipogenic genes involved in de novo lipogenesis and facilitates the esterification of free fatty acids to triglycerides controlled by PKCζ-induced sterol regulatory element binding transcription factor 1 (Srebf1). Taken together, ceramide modulates many of the lipid regulatory factors and lipogenic enzymes and, in doing so, augments intracellular triglyceride accumulation.

Mitochondria is the organelle that contains various enzymes involved in ceramide metabolism. In particular, CerS5 and CerS6 have been identified in mitochondria-associated membranes and mitochondria [29]. Mitochondrial CerS6 expression was increased in response to diet-induced obesity [29]. Functionally, elevated C16:0-ceramides impair fatty acid oxidation by inactivating the electron transport chain and increase reactive oxygen species [73]. Similarly, increased intracellular ceramides via knocking out sphingomyelin synthase 2 impairs mitochondrial respiration in cultured myotubes [74]. In addition, CerS6-derived C16:0-ceramides promote mitochondrial fragmentation and impair mitochondrial function via binding of the mitochondrial fission factor in obese mice [29]. Furthermore, CerS1-derived C18:0-ceramides promotes lethal mitophagy, resulting in reduced mitochondrial fatty acid oxidative capacity [75]. Altogether, ceramides impair mitochondrial dynamics and function which have been implicated in obesity and related metabolic diseases.

## 4. Impact of Bioactive Phytochemicals on Ceramide Metabolism and Obesity-Related Metabolic Diseases

We focus on the effects of phytochemicals and their rich dietary sources on ceramide levels as well as the progression of obesity and metabolic diseases. Diverse phytochemicals are covered in this paper to show the overarching interest in phytochemical studies, all of which can be classified as (1) alkaloids (caffeine and its derivative); (2) sulfur-containing compounds (sulforaphane); (3) stilbens (resveratrol); and (4) flavonoids (chamiloflan, proanthocyanidin, catechin, anthocyanin, and xanthohumol). We review in vitro, in vivo, and human clinical/epidemiological studies under obesity, metabolic disease-related pathological conditions, as well as physiological normal condition (Table 1 and Table 2). In addition to obesity and related metabolic diseases, various phytochemicals are able to alleviate the development of cancer via modulating ceramide levels [76]; however, this topic is beyond our purpose, and we exclude the studies using cancer-related models in this paper.

### 4.1. Coffee, Caffeine, and Caffeine Derivative

Coffee, a heterogenous mixture containing hundreds of phytochemicals, is one of the most frequently consumed beverages. Accumulating evidence suggests that coffee consumption has been consistently associated with a reduced risk of chronic diseases including cardiovascular diseases [81]. Signori et al. reported that in patients with symptomatic chronic heart failure, higher coffee consumption was associated with a lower risk of incident atrial fibrillation, a major cardiovascular event [82]. This study also showed that higher coffee consumption was associated with elevated concentrations of plasma C24:0 ceramides (d18:1/24:0), which may suggest a plausible mechanism of the inhibitory effect of caffeine on CVD. Similarly, Seow et al. reported that long-term coffee consumption was significantly associated with high levels of plasma sphingolipids including 32 ceramides including C24:0-ceramides and 29 sphingomyelins in a Singapore population [83]. In addition, Wittenbecher et al. showed that coffee consumption was associated with lower concentrations of C22:2-dihydroceramide, which is associated with both T2DM and CVD risks in the prospective EPIC-Potsdam study using mediation analyses [84]. Collectively, these human studies suggest that coffee consumption might have beneficial effects on cardiometabolic diseases via mediating (dihydro)ceramide levels.

Caffeine is a major bioactive compound in coffee known to yield various health outcomes. The above-mentioned human studies only considered dietary patterns, not specific information regarding coffee composition such as type of coffee bean, preparation methods, and other additives (e.g., milk and sugar). Rodent studies enabled the investigation of the potential effects of pure caffeine in ceramide metabolism. Sinha et al. demonstrated that caffeine treatment alleviated NAFLD by increasing hepatic autophagy using in vitro and mice models [85]. Intraperitoneal injection of caffeine (30 mg/kg BW equivalent to two–three cups of coffee in humans) has shown to decrease levels of ceramides (C22:0-, C25:0-) and dihydroceramide (C22:0-) but increase levels of sphingosine in the livers of normal fat diet-fed mice. However, this study only used normal physiological mice, not those in an HFD-induced, metabolically dysfunctional state. Similarly, Velázquez et al. showed the effect of the supplementation of polyphenol-rich green coffee extract (0.18 g of caffeine/kg diet equivalent to one cup of coffee in humans), and caffeine alone (0.18 g/kg diet) in female Sprague–Dawley rats fed a high-fat, high-fructose diet (HF-HFr) [86]. Green coffee extract alleviated hepatic steatosis without changing body weight and inflammation; caffeine did not. Caffeine and green coffee extract supplementation significantly reduced hepatic ceramides (C18:1- and C20:0-) compared to the HF-HFr group.

Caffeic acid phenethyl ester (CAPE) is a coffee phenolic compound and also a main bioactive compound in propolis extract. Zhong et al. demonstrated that 8 weeks of CAPE treatment (75 mg/kg/d) ameliorated HFD-induced obesity and hepatic steatosis [87]. The authors mechanistically revealed that CAPE treatment significantly reduced serum and ileum ceramide levels via inhibiting bacterial bile salt hydrolase, modulating bile salt, and then inhibiting the FXR signaling pathway. Additionally, it was shown that the effects of CAPE on hepatic steatosis were removed in the intestinal FXR deficient mice. As a result, consumption of coffee, caffeine, or its derivative has a potential to modulate ceramide metabolism, and subsequently contribute to alleviate the development of obesity and hepatic steatosis.

### 4.2. Sulforaphane

Cruciferous vegetables such as broccoli, cabbage, and brussels sprouts are rich sources of glucosinolates, a sulfur-containing compound. Sulforaphane (SFN) is a bioactive isothiocyanate derived from glucosinolate. Accumulating clinical studies have shown that consumption of SFN-rich diets can improve lipid profiles and glucose control [88,89]. Similarly, a meta-analysis study demonstrated that SFN supplementation can reduce body weight and decrease circulating total cholesterol, LDL-cholesterol, and triglyceride levels in rodents [88]. Although SFN supplementation has shown the capability of improving lipid profiles, few studies investigated the effects of SFN in ceramide accumulation. Teng et al. found that SFN treatment (10 μM) significantly reduced total ceramide levels in palmitate-treated HepG2 cells via decreasing de novo synthetic pathway (decreased *CerS2*, *CerS4*, *Sptlc3*) [90]. Consistently, SFN administration (0.5 mg and 5 mg SFN/kg, i.p. injection three times a week) significantly decreased hepatic ceramide accumulation in HFD-fed mice. The authors also demonstrated that SFN supplementation improved glucose tolerance and insulin sensitivity in mice. Li et al. corroborated the effect of SFN both in PA-treated HepG2 cells and in the livers of HFD-fed mice [91]. Moreover, SFN administration (10 mg SFN/kg/d, i.p.) alleviated HFD-induced body weight gain, insulin resistance, and hepatic steatosis in mice. Together, these two studies consistently showed the effects of SFN in decreasing hepatic ceramide accumulation in both in vitro and in vivo models. More studies are needed to clarify the underlying mechanism of SFN’s effects and also confirm this in human subjects.

### 4.3. Resveratrol

Resveratrol, a non-flavonoid polyphenol found in skin and seeds of grapes and red wine, has potential health benefits such as antiaging, antidiabetic, and cardioprotective activities [92]. Evidence shows that resveratrol can exhibit various metabolic benefits by modulating molecular targets such as sirtuin 1 (SIRT1) and AMP-activated protein kinase (AMPK) [93]. However, its effects on ceramide metabolism are only partially understood. Bikman et al. showed that resveratrol treatment (20 μM) inhibited palmitate-induced ceramide accumulation (total Cer, d18:1/16:0), as well as improved insulin signaling in cultured myotubes [77]. The authors found that these effects were independent of SIRT1 signaling. Similarly, Momchilova et al. showed that resveratrol treatment (50 μM) significantly reduced ceramide levels by activating neutral sphingomyelinase (nSMase) in plasma membranes of hepatocytes isolated from aged rats though not in metabolic disease model [78]. Furthermore, Alrob et al. demonstrated the effect of resveratrol administration in mice [94]; BALB/c mice were fed high-fat diet (HFD) for 8 weeks, followed by either HFD, HFD with i.p. injection of resveratrol (30 mg/kg BW), or LFD with i.p. injection of resveratrol for 4 weeks. Here, the authors found that muscle ceramide levels were significantly reduced in mice treated with LFD and resveratrol but not with HFD and resveratrol compared to HFD-fed mice. Both resveratrol treatment groups have shown improved insulin sensitivity and secretion compared to the HFD group. This study showed the beneficial effect of resveratrol in muscle ceramide levels, but the resveratrol-mediated ceramide metabolism needs to be understood. Altogether, these findings highlight the potential role of resveratrol on ceramide metabolism; however, more in vivo and clinical studies are needed to corroborate this finding.

**Table 2:** Summary of animal studies examining the impact of phytochemical consumption on sphingolipid levels and related metabolism.

Author	Animal Model	Treatment	Duration	Ceramide Levels	Sphingolipids Levels	Sphingolipid Metabolism-Related Expression or Activity
Sinha et al. [85]	C57BL/6 mice, ♂	Caffeine (30 mg/kg BW) I.P. injection + ND vs. Untreated + ND	3 d	↓ Hepatic C22:0-, C25:0-Cer ∅ Hepatic C2:0-, C16:0-, C18:0-, C20:0-, C22:1-, C23:0-, C24:0-, C24:1-Cer	↓ Hepatic C22:0-DhCer ∅ Hepatic C16:0-, C24:0-, C24:1-DhCer ↑ Hepatic Sphiganine, Sphingosine	-
Velázquez et al. [86]	Sprague Dawley rats, ♀	Caffeine + HF-HFr vs. GCE + HF-HFr vs. HF-HFr	HF-HFr for 2 mo + Additional treatment for 1 mo	Caffeine: ↓ Hepatic 18:1-Cer ∅ Hepatic C14:0-, C16:0-, C18:0-, C20:0-, C22:0-, C24:0-, C24:1-Cer GCE: ↓ Hepatic 20:0-Cer ∅ Hepatic C14:0-, C16:0-, C18:0-, C22:0-, C24:0, C18:1-Cer	Caffeine: ∅ Hepatic C16:0-, C18:0-, C20:0-, C22:0-, C24:0-, C24:1-HexCer GCE: ↓ Hepatic 18:0-, 20:0-, 22:0-HexCer ∅ Hepatic C16:0-, C24:0-, C24:1-HexCer	-
Zhong et al. [87]	C57BL/6 mice, ♂	CAPE (75 mg/kg/d) + HFD vs. HFD	8 wk	-	-	↓ Ileum *Sptlc2* *CerS2* *CerS4*
	FXR^fl/fl^ FXR^ΔIE^, ♂	CAPE (75 mg/kg/d) vs. saline	8 wk	CAPE: ↓ Serum total-Cer ↓ Ileum total-, C16:0-,C18:0-,C20:0-,C22:0-, C24:0-Cer	-	-
Teng et al. [90]	C57BL/6J mice, ♂	SFN (0.5 mg/kg, 5 mg/kg, 3 times/wk i.p.) + HFD vs. HFD	10 wk	↓ Hepatic total-Cer	-	↓ Hepatic *Sptlc3* *CerS4*
Li et al. [91]	C57BL/6J mice, ♂	SFN (10 mg/kg/d, i.p.) + HFD vs. HFD	17 wk	↓ Hepatic total-Cer	-	-
Alrob et al. [94]	BALB/c mice, ♂	RES (30 mg/kg, every other day i.p.) + LFD vs. HFD	4 wk HFD + 4 wk treatment	↓ Muscle total-Cer	-	-
Babenko et al. [79]	Wistar rat, ♂	Chamiloflan (160 mg/kg BW, daily i.p.)	1 wk	3-mo-old: ∅ Hepatic total-Cer 24-mo-old: ↓ Hepatic total-Cer 27–28-mo-old: ↓ Hepatic total-Cer	3-mo-old: ∅ Hepatic SM 24-mo-old: ↑ Hepatic SM 27–28-mo-old: ↑ Hepatic SM	24-mo-old: ↓ nSMase activity
Tveter et al. [95]	*db/db* mice	LFD (10% SPI) with 1% GP vs. LFD (10% SPI)	28 d	-	-	↓ Hepatic *Sptlc2* *CerS4* *Fxr* ↓ Ileum *Smpd3* *Fxr*
Seo et al. [96]	C57BL/6J mice, ♂	ChrSd (10% *w*/*w*) + HFD vs. HFD	5 wk	-	-	↓ Hepatic Sptlc3 (mRNA, protein)
Cho et al. [97]	C57BL/6J mice, ♂	GSF (10% *w*/*w*) + HFD vs. HFD	5 wk HFD + 9 wk treatment	-	-	↓ Intestinal *Fxr* ∅ Adipose tissue
Huang et al. [98]	C57BL/6J mice, ♂	EGCG (3.2 g/kg diet) + HFD vs. HFD	17 wk	↓ Hepatic d18:1/16:0-, d18:1/26:0- d18:1/26:1-Cer ↑ Hepatic d18:1/18:0-, d18:1/22:1-, d18:1/24:2-Cer ↓ Serum d18:1/16:0-, d18:1/22:3-Cer	↓ Hepatic d18:1/18:3-SM ↑ Hepatic d18:1/20:0-, d18:1/22:0-, d18:1/22:1-, d18:1/24:0-, d18:1/24:1-, d18:1/24:2-, d18:1/24:3-, d18:1/26:3-, d18:1/26:4-SM ↑ Serum d18:1/16:0-, d18:1/18:1-, d18:1/18:3-, d18:1/20:0-, d18:1/22:0-, d18:1/22:1-, d18:1/24:0-, d18:1/24:3-SM	-
Nam et al. [99]	C57BL/6J mice, ♂	Green tea extract (0.25% *w*/*w*) + HFD vs. HFD	12 wk	↑ Hepatic d18:1/22:0-Cer	-	-
Ali et al. [100]	Sprague–Dawley rats, ♂	cocoa polyphenol (600 mg/kg BW/d) + HFD vs. HFD	HFD for 12 wk + Treatment for 4 wk	-	-	↓ MES-WAT *Cers5 Fa2h*
Si et al. [101]	C57BL/6 mice, ♂	BAE (200 mg/kg BW) + HFD vs. HFD	8 wk	↓ Serum Total-Cer All examined Cer	↓ Serum SM	↑ Serum *SMS1, SMS2* ↓ Serum *Spt*, *CerS1, CerS2*, *CerS4*, *Degs, ASMase*
Paraiso et al. [102]	C57Bl/6J WT, FXR^Liver−/−^ mice, ♂ ♀	XN (60 mg/kg BW/d) + HFD vs. HFD	12 wk	XN (WT, ♀): ↓ Hepatic total-Cer XN (WT, ♂): ∅ Hepatic total-Cer XN (FXR^Liver−/−^, ♀): ∅ Hepatic total-Cer XN (FXR^Liver−/−^, ♂): ↓ Hepatic total-Cer	XN (WT, ♀): ↓ Hepatic SM XN (WT, ♂): ↑ Hepatic SM XN (FXR^Liver−/−^, ♀): ∅ Hepatic SM XN (FXR^Liver−/−^, ♂): ∅ Hepatic SM	-
Paraiso et al. [103]	C57Bl/6J mice, ♂	XN +HFD DXN HFD TXN + HFD (each flavonoid dose, 30 mg/kg BW per day) vs. HFD	13 wk	XN, DXN, TXN: ↓ Hepatic total-Cer ↓ Hippocampal total- Cer	XN, DXN, TXN: ∅ Hepatic total SM	XN: ↑ Hepatic *Degs2*, *Cers2,4,5,6, Smpd1,3,4*, *Sgms1,2* DXN, TXN: ↑ Hepatic *Sptlc1, Smpd4*

Abbreviations: ASMase, sphingomyelin phosphodiesterase; BAE, Blueberry anthocyanin-rich extract; CAPE, caffeic acid phenethyl ester; Cer, ceramide; Cers, ceramide synthase; ChrSd, Chardonnay grape seed flour; DAG, diacylglycerol; DEGS, sphingolipid 4-desaturase; DhCer, dihydroceramide; DXN, dihydroxanthohumol; EGCG, epigallocatechin-3-gallate; Fa2h, fatty acid 2-hydroxylase; FXR, farnesoid X receptor; GCE, green coffee extract; GSF, grape seed flour; HFD, high-fat diet; HexCer, hexosylceramide; HF-HFr, high-fat-high-fructose group; i.p., intraperitoneal; LFD, low fat diet; MES-WAT, mesenteric white adipose tissue; ND, normal-fat diet; RES, resveratrol; SFN, sulforaphane; Sgms, sphingomyelin synthase genes; SM, sphingomyelin; SPI, soy protein isolate; Smpd, sphingomyelin phosphodiesterase; SMS, sphingomyelin synthase; SPT, serine palmitoylotransferase; Sptlc, serine palmitoyltransferase long chain base subunit; TXN, tetrahydroxanthohumol; XN, xanthohumol; ∅, no change; ↓, decrease; ↑, increase; ♂, male; ♀, female.

### 4.4. Tea Flavonoids and Chamiloflan

Tea is a flavonoid-rich food. Along with coffee, tea is one of the most popular beverages worldwide. Tea consumption shows positive health effects such as reduced risks of total mortality, cardiovascular diseases, and type 2 diabetes in a dose-dependent manner based on a recent meta-analysis study [104]. Unlike coffee consumption, there was no significant association between green tea consumption and circulating ceramides in Asian populations [83]. This study revealed that black tea consumption is significantly associated with higher levels of nine metabolites, including sphingomyelin, sphingosine-1-phosphate, and dihexosylceramide, and green tea consumption is significantly inversely associated with four metabolites including sphingomyelin.

Babenko et al. explored the effects of chamomilla recruita flavonoids (chamiloflan; a mixture of apigenin, luteolin, apigenin-7-glucoside, luteolin-7-glucoside, isorhamnetin, and quercetin) in the aged rat liver and hepatocytes [79]. Total ceramide deposition in the liver was elevated in the aged liver compared to the young liver, which is often shown during aging process. Intragastric administration of chamomile flavonoids (160 mg/kg BW) significantly reduced hepatic ceramide accumulation via increasing the activities of neutral and acid SMases. Specifically, treatment of a single chamiloflan component, apigenin-7-glucoside, significantly reduced intracellular ceramide levels in the isolated hepatocytes from 24-month-old rats compared with the control. Another study by Babenko et al. also demonstrated the effects of chamiloflan in the CCl_4_- or ethanol-damaged rat liver and hepatocytes [80]. Chamiloflan significantly reduced CCl_4_- or ethanol-induced ceramide accumulation in the rat liver and hepatocytes.

### 4.5. Grape Seed and Proanthocyanidin

Proanthocyanidins are the oligomers or polymers of flavano-3-ol, rich in grape seeds, cranberries, and pomegranates. Proanthocyanidins are known to exert beneficial health effects including modulating gut microbiota as prebiotics [105]. Only few rodent studies showed the effects of proanthocyanins on ceramide metabolism.

Tveter et al. examined the role of proanthocyanidin-rich grape polyphenols (GP, 1%) in leptin-deficient *db/db* mice [95]. GP supplementation significantly decreased mRNA expression of genes involved in ceramide synthesis in the liver (*Sptlc2, CerS4*) and ileum (*Smpd3*), suggesting that GP may decrease tissue ceramide accumulation. Of note, the authors mechanistically demonstrated that GP remodeled gut microbial composition and diversity and significantly reduced *Fxr* expression compared to the control, suggesting that GP altered ceramide metabolism via gut microbiota-BA-FXR signaling pathways. Seo et al. investigated the effects of flavonoid-rich Chardonnay grape seed flour (ChrSd; 10%) in HFD-fed mice [96]. ChrSd supplementation alleviated obesity and related insulin resistance, and hepatic steatosis in HFD-fed mice. Authors noticed that ChrSd supplementation significantly decreased hepatic expression of *Sptlc3*, which might indicate decreased ceramide de novo synthesis. However, it was not confirmed by hepatic ceramide levels in these mice. Similarly, Cho et al. demonstrated that grape seed flour (GSF; 10%) alleviated obesity and glucose homeostasis in HFD-fed obese mice [97]. Interestingly, GSF showed downregulated intestinal *Fxr* expression that can plausibly modulate ceramide accumulation. However, this study showed that GSF did not change gene expression related to ceramide metabolism in the adipose tissue based on microarray. Additionally, this study showed that the combined GSF with lactic acid bacteria significantly reduced gene expression involved in ceramide de novo synthesis in the adipose tissue.

Furthermore, Prasain et al. reported that proanthocyanidin-rich cranberry supplementation (1 g/kg BW) reduced plasma C20:0-ceramide in HFD-fed rats in the unpublished work [106]. Altogether, proanthocyanidins have shown the potential to decrease ceramide accumulation in HFD-induced obese mice via modulating the BA-FXR signaling pathway. However, more clinical studies are needed for confirmation in the future.

### 4.6. Green Tea, Cocoa, and Catechin

The catechin family represents the compounds derived from catechin, including epigallocatechin-3-gallate (EGCG), epigallocatechin (EGC), epicatechin, epicatechin-3-gallate, gallocatechin gallate (GCG), and gallocatechins. Catechin flavonoids are the main compound in green tea, and are also found in cocoa and berries. Accumulating studies have shown that consumption of catechin-rich green tea could have health-promoting effects such as reducing the risk of obesity and cardiovascular disease [107,108]. Catechin-rich cocoa polyphenols also have shown many beneficial effects, primarily in cardiovascular diseases [109]. Many studies reported the impact of catechin supplementation or catechin-rich food in ceramide accumulation as well as the progression of obesity and related metabolic diseases.

A cohort study demonstrated that green tea consumption was not associated with circulating ceramides in Asian populations [83]. In contrast, rodent studies have shown the potential of catechins and catechin-rich foods to alter ceramide accumulation in the metabolically active tissues. Nam et al. reported that 0.25% green tea extract supplementation alleviated HFD-induced obesity and hepatic steatosis in mice [99]. This study revealed that hepatic ceramide (d18:1/22:0) levels were lowered by HFD and restored by green tea extract supplementation. Huang et al. reported that three ceramide species (d18:1/16:0, d18:1/26:0, d18:1/26:1) were decreased but three ceramide (d18:1/18:0, d18:1/22:1, d18:1/24:2) species were increased by EGCG supplementation (3.2 g/kg diet) in HFD-fed mice [98]. Of note, EGCG supplementation lowered lipid absorption by modulating bile acid homeostasis, which alleviates HFD-induced hepatic steatosis and metabolic dysfunctions. However, the alteration of specific ceramide species in opposite directions via EGCG needs to be elucidated.

Ali et al. investigated the effect of cocoa polyphenol treatment (600 mg/kg bw/day) in HFD-induced obese rats [100]. Here, cocoa polyphenols contained mainly five polyphenolic molecules—gallic acid, protocatechuic acid, chlorogenic acid, epicatechin, and catechin. Cocoa polyphenol treatment alleviated HFD-induced obesity and dyslipidemia. In addition, DNA microarray analysis in mesenteric white adipose tissue revealed that 30 genes including ceramide synthase 5 were down-regulated in the HFD supplemented with cocoa powder, as compared with the HFD-fed group. Though the authors did not report ceramide accumulation in tissues, this study suggests a possibility that cocoa polyphenol treatment might lead to decrease ceramide levels in white-adipose tissue by decreasing ceramide biosynthesis.

Kobayashi et al. investigated the mechanisms of catechin-mediated ceramide alteration, specifically whether various catechins and their derivatives attenuate the secretory sphingomyelinase activity in rat plasma [110]. Secretory SMase, a product of the acid SMase gene, is found in extracellular environments. Authors demonstrated that 50 μM catechins ((-)-epicatechin-3-gallate, (-)-epigallocatechin-3-gallate (EGCG), (-)-catechin-3-gallate) and methylated (-)-epicatechin 3-gallate molecules decreased activities of secretary SMase in rat plasma. Although the authors did not report a change in circulating ceramide levels, this study suggests the possibility that catechin treatment might lead to decrease the circulating ceramide levels by decreasing secretory SMase activity.

Altogether, catechin and catechin-rich foods have shown the effect of modulating ceramide accumulation in the liver and adipose tissues, potentially via altering ceramide synthesis and degradation of sphingomyelin. Future studies need to clarify the mechanism of which catechin supplementation alters specific ceramide species in opposite directions.

### 4.7. Anthocyanins

Anthocyanins, one group of flavonoids, are water-soluble pigments that confer the red, blue, and purple colors to various fruits and vegetables such as berries, beets, grapes, and currants. Anthocyanins have gained their attention due to their potential therapeutic effects on human health [111]; however, only a few studies have investigated whether anthocyanin consumption is associated with ceramide metabolism. Si et al. fed mice a high-fat diet supplemented with blueberry anthocyanin-rich extract (100 mg/kg or 200 mg/kg) for 8 weeks [101]. High dose of anthocyanins has been shown to reduce all species of ceramides in serum by suppressing de novo synthesis and enhancing salvage pathway. In addition, the authors showed that anthocyanin supplementation reduced body weight and improved insulin signaling and liver function. In particular, anthocyanin supplementation significantly reduced mRNA levels of PP2A and PKCζ, which are the targets for ceramides. A recent randomized controlled trial in dyslipidemia subjects showed that 12 weeks of consuming dietary anthocyanins reduced plasma C16:0- and 18:0-ceramides in a dose-dependent manner [112]. Together, these studies suggest the potential role of dietary anthocyanin in regulating circulating ceramide levels, and subsequently attenuating metabolic diseases.

### 4.8. Xanthohumol

Xanthohumol (XN) is the most abundant prenylated flavonoid found in hops. Paraiso et al. conducted a study in which mice consumed HFD containing either XN or its hydrogenated derivatives, dihydroxanthohumol (DXN) and tetrahydroxanthohumol (TXN) (30 mg/kg BW per day, respectively), for 13 weeks [103]. Each hop-derived flavonoid supplementation alleviated obesity-induced insulin resistance and cognitive impairment in HFD-fed mice, which has been consistently reported in HFD-fed rodents [113,114,115]. XN and its derivatives decreased total ceramide levels in the liver and hippocampus of HFD-fed mice. There was a significant strong correlation between total ceramide levels in the hippocampus and cognitive performance. Furthermore, XN and its derivatives differentially modulated the expression of genes involved in ceramide metabolism in the liver. In particular, XN increased genes related to ceramide synthesis, sphingomyelinase, and sphingomyelin synthase, suggesting an increased turnover of ceramides in the liver. In addition to ceramides, hop-derived flavonoids significantly decreased other lipid species in the liver including cholesterol, and TG via activating FXR target genes. Similarly, Paraiso et al. also showed that XN supplementation (60 mg/kg BW per day) for 12 weeks significantly decreased the proportion of ceramide over total sphingolipids in male WT and liver-specific FXR-null (FXR^liver−/−^) mice, but not in female mice [102]. Interestingly, XN supplementation induced CAR, PXR, and GR as complementary regulation in FXR^liver−/−^ mice, which alleviated dysregulated lipid metabolism. Together, hop-derived flavonoids have been shown to decrease peripheral ceramide levels along with other lipid species, suggesting a potential role in preventing obesity-related metabolic diseases.

## 5. Conclusions

**Figure 2:**
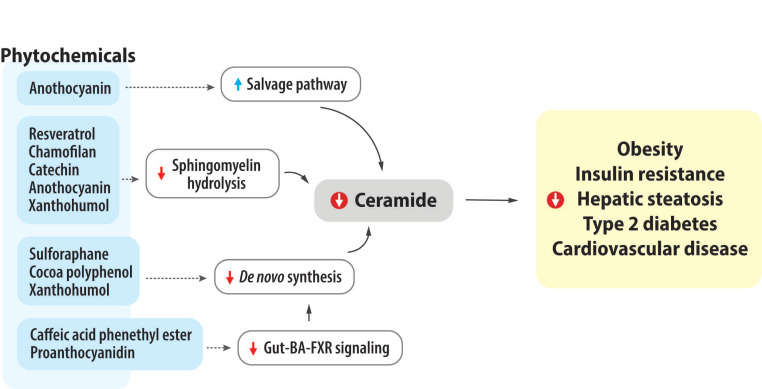
The impact of phytochemicals on ceramide metabolism and physiological results.

Ceramide is an important and deleterious lipid signal that can contribute to the development of various obesity-related metabolic diseases. Remarkably, ceramides appear to be a therapeutic target and biomarker for these diseases. In particular, ceramide levels can be either exacerbated or improved by diets, and phytochemicals are a good candidate for reducing ceramide deposition in body compartments. In this review, we revealed that numerous phytochemicals are able to alter ceramide accumulation, and then reduce the risk of obesity-related metabolic diseases. The mechanisms of changing ceramide levels are diverse and depend upon the types of phytochemicals; for example, reducing ceramide de novo synthesis, suppressing sphingomyelin hydrolysis, increasing salvage pathway, or changing gut-bile acid-FXR axis (Figure 2). In conclusion, consumption of phytochemicals has the potential to prevent or reverse obesity and related metabolic diseases.

We demonstrated that there is a wide knowledge gap regarding the role of phytochemicals in ceramide metabolism associated with obesity-related metabolic diseases. Many of the reviewed studies only considered the effects of phytochemicals on ceramide profiles in the body; they did not explore the associated enzymes or signaling pathways, which need to be clarified in the future. Among the studies, changes in specific ceramide species after phytochemical supplementation also vary among tissues. Lastly, more studies need to explore a variety of individual phytochemicals and their dietary sources, which will aid in developing specific dietary regimens to lower ceramides and improve patient health.

## Figures and Tables

**Table 1:** Summary of in vitro studies examining the impact of phytochemical treatment on sphingolipid levels and related metabolism.

Author	Cells	Treatment	Ceramide/Sphingolipid Levels	Sphingolipid Metabolism-Related Expression or Activity
Bikman et al. [77]	C2C12 (myoblasts)	20 μM RES + 0.75 mM PA vs. 0.75 mM PA	↓ d18:1/16:0-, Total-Cer ↑ d18:1/16:0-, Total-DhCer	-
Momchilova et al. [78]	Hepatocytes from 20-mo-old Wistar rats (♂)	50 μM RES vs. CON	↑ SM	↓ nSMase activity
Babenko et al. [79]	Hepatocytes from 24-mo-old Wistar rats (♂)	500 μg/mL Chamiloflan 30 μM AP7Glu 30 μM LU7Glu vs. CON	Chamiloflan: ∅ Total-Cer AP7Glu: ↓ Total-Cer LU7Glu: ∅ Total-Cer	↓ nSMase activity
Babenko et al. [80]	Hepatocytes from 90-d-old Wistar rats (♂)	Pretrt 40 mM CCl_4_ + 500 μg/mL Chamiloflan Pretrt CCl_4_ + 30 μM AP7Glu vs. CCl_4_	Chamiloflan: ↓ Total-Cer, ↑ SM AP7Glu: ↓ Total-Cer, ∅ SM	-
		Pretrt 70 mM EtOH + 500 μg/mL Chamiloflan vs. EtOH	Chamiloflan: ∅ Total-Cer, ∅ SM	

Abbreviations: AP7Glu, apigenin-7-glucoside; Cer, ceramide; CON, control; DhCer, dihydroceramide; ethanol, EtOH; LU7Glu, luteolin-7-glucoside; nSMase, neutral sphingomyelinase; PA, palmitate; Pretrt, pretreatment; RES, resveratrol; SM, sphingomyelin; ∅, no change; ↓, decrease; ↑, increase; ♂, male.

## Data Availability

No new data were created or analyzed in this study.
